# USE OF A NOVEL STAFFING PROGRAM IN NURSING HOMES: A MIXED METHODS STUDY

**DOI:** 10.1093/geroni/igae098.0225

**Published:** 2024-12-31

**Authors:** Lindsay Peterson, Kelly Smith

**Affiliations:** University of South Florida, Tampa, Florida, United States; University of South Florida, Tampa, Florida, United States

## Abstract

Staffing in nursing homes (NHs) has long been a critical policy issue. Faced with NH staffing challenges, Florida legislators approved a 2021 measure allowing lesser trained staff (personal care attendants [PCAs]) to work as certified aides for four months, with the expectation the PCA would become certified in that time. We used mixed methods, aiming to assess Florida NH administrators’ views on this novel staffing program. We distributed a Qualtrics survey of closed- and open-ended questions to 695 administrators via email between July 2022 and September 2022, receiving 55 unduplicated, complete responses. Logistic regression was used to estimate relationships between independent variables (e.g. facility characteristics, federal five-star rating, survey responses) and the outcome, whether respondents found the PCA program beneficial (yes/no). To build on quantitative results, we used thematic analysis to assess four open-ended survey questions. Results found the average bed size of respondents’ NHs was 135, 75% were nonprofit and 62% were part of a chain. Regression results found a significant relationship between rating the program as beneficial and hiring more PCAs (p=.003) and having a higher quality rating (p=.02). We identified three qualitative themes, 1) benefits of the program, 2) barriers to success, 3) how to make the program work, with an overarching theme of administrative proactivity. Analysis of quantitative and qualitative results together found administrators who rated the PCA program as beneficial were more likely to describe proactive efforts (e.g. establishing PCA mentorship programs). Results provide guidance on practices required to make a novel staffing program succeed.

